# Supplementary material for: Genomic diversity and novel genome-wide association with fruit morphology in Capsicum, from 746k polymorphic sites
Source: Sci Rep. 2019 Jul 11;9:10067. doi: 10.1038/s41598-019-46136-5 (PMC6624249; doi:10.1038/s41598-019-46136-5)
Supplement: Supplementary file 1 — Supplementary figures [file 41598_2019_46136_MOESM1_ESM.pdf]

Supplementary Information for: Genomic diversity and novel genome-wide association with fruit morphology in *Capsicum*, from 746k polymorphic sites.

Vincenza Colonna[1,*], Nunzio D'Agostino[2], Erik Garrison[3], Anders Albrechtsen[4], Jonas Meisner[4], Angelo Facchiano[5], Teodoro Cardi[2], and Pasquale Tripodi[2,*]

[1]Institute of Genetics and Biophysics, National Research Council (CNR), Naples (Italy)
[2]CREA Research Centre for Vegetable and Ornamental Crops, Pontecagnano Faiano (Italy)
[3]Wellcome Sanger Institute, Hinxton (UK)
[4]Bioinformatics Center, Department of Biology, University of Copenhagen (Denmark)
[5]Institute of Food Science, National Research Council (CNR), Avellino (Italy)
[*]vincenza.colonna@igb.cnr.it, pasquale.tripodi@crea.gov.it

ABSTRACT

Capsicum is one of the major vegetable crops grown world-wide. Current subdivision in clades and species is based on morphological traits and coarse sets of genetic markers. Fruits broad variability has been driven by breeding programs and has been mainly studied by linkage analysis.
We discovered 746k variable sites by sequencing 1.8% of the genome in a collection of 373 accessions belonging to 11 *Capsicum* species from 51 countries. We describe genomic variation at population-level, confirm major subdivision in clades and species, and show that the known major subdivision of *C. annuum* separates large and bulky fruits from small ones. In *C. annuum*, we identify four novel loci associated with phenotypes determining the fruit shape, including a non-synonymous mutation in the gene *Longifolia 1-like* (CA03g16080).
Our collection covers all the economically important species of *Capsicum* widely used in breeding programs, and represent the widest and largest study so far in terms of the number of species and number of genetic variants analyzed. We identified a large set of markers that can be used for population genetic studies and genetic association analyses. Our results provide a comprehensive and precise perspective on genomic variability in *Capsicum* at population-level and suggest that future fine genetic association studies will yield useful results for breeding.

Figure S1. Size in nucleotides of deletions and insertion (InDel). Due to our use of GBS and reference-guided analysis, we were only able to discover InDels up to a few tens of bases. InDels of three or multiples of three nucleotides seems more frequent in genic region compared to intergenic ones, suggesting a preference for InDels that add or remove triplets over those causing frame-shifts mutations.

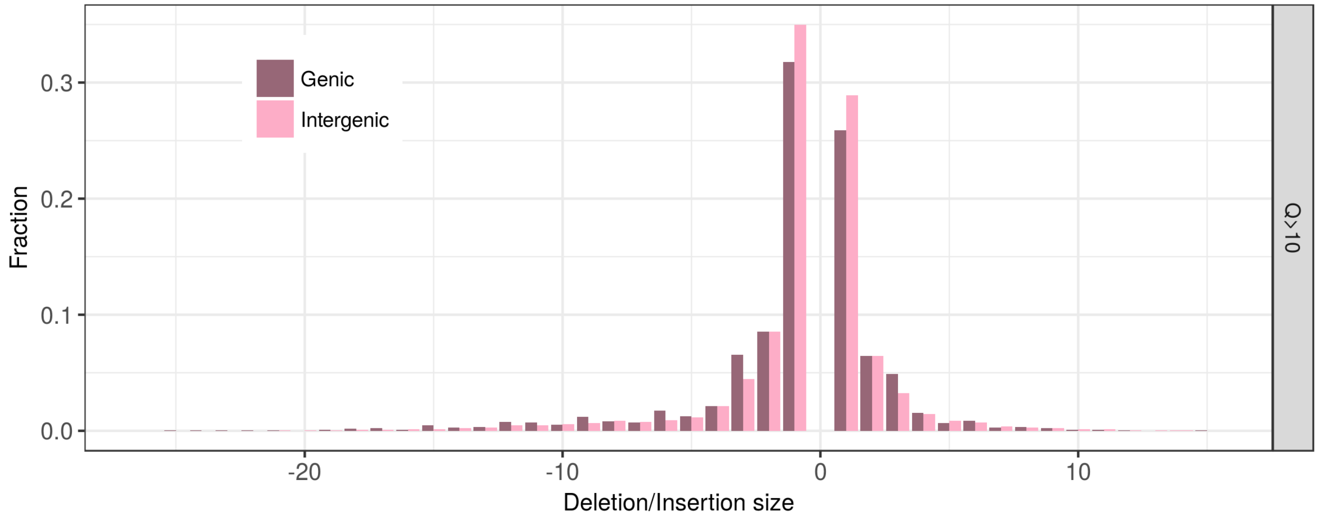

Supplementary Figures

Figure S2. **Principal component analysis (PCA) based on 746k genetic variants.** Colors reflect the geographical origin of the accessions

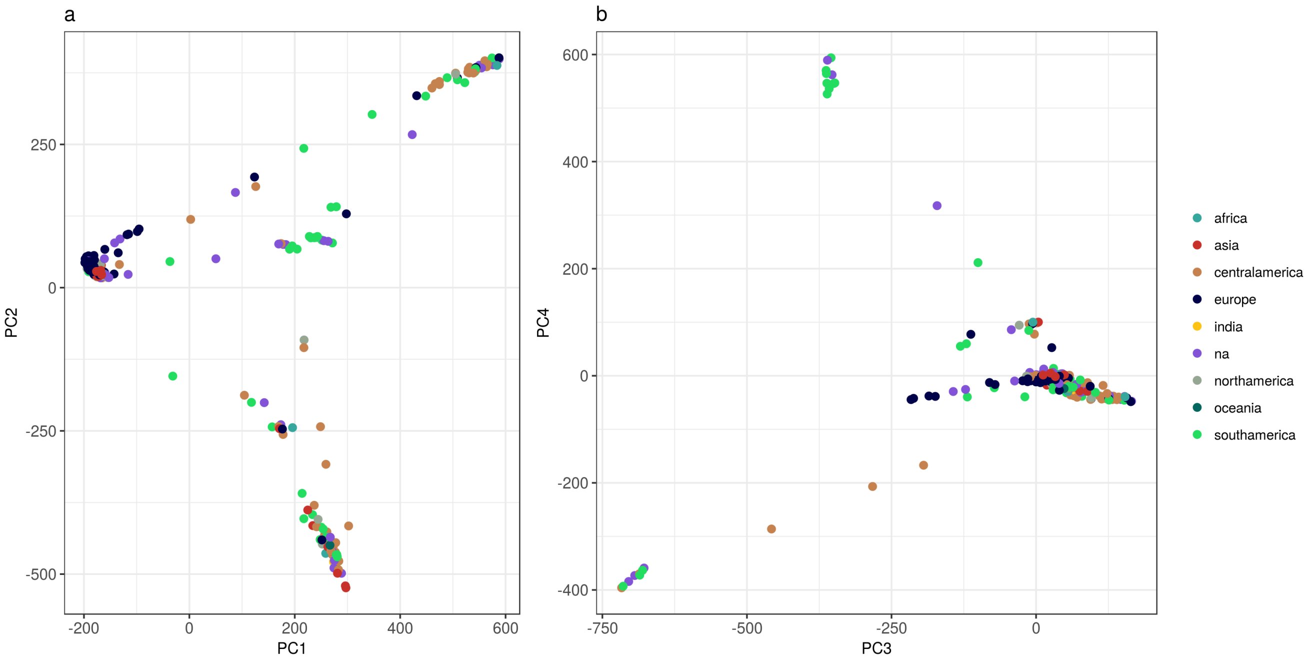

Figure S3. **Cross-validation error of the admixture estimate for the hypotheses of 2 to 10 clusters**

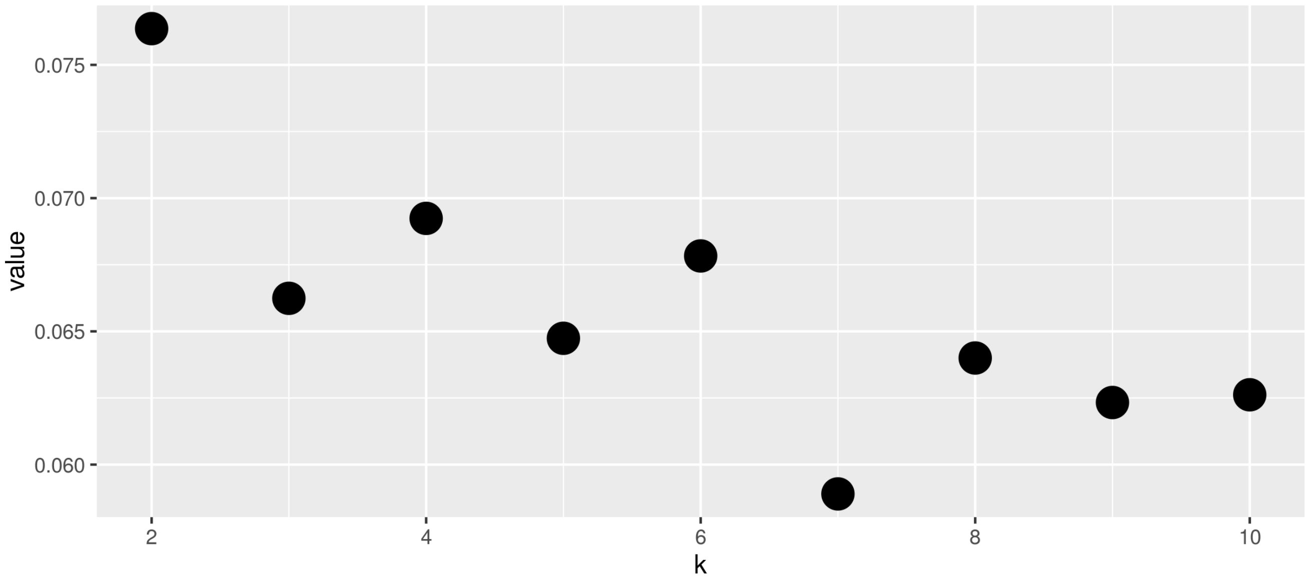

Figure S4. Admixture analysis. In the ADMIXTURE analysis most accessions belong to only one cluster, with the median coefficient of membership to the best-matching cluster being 0.99

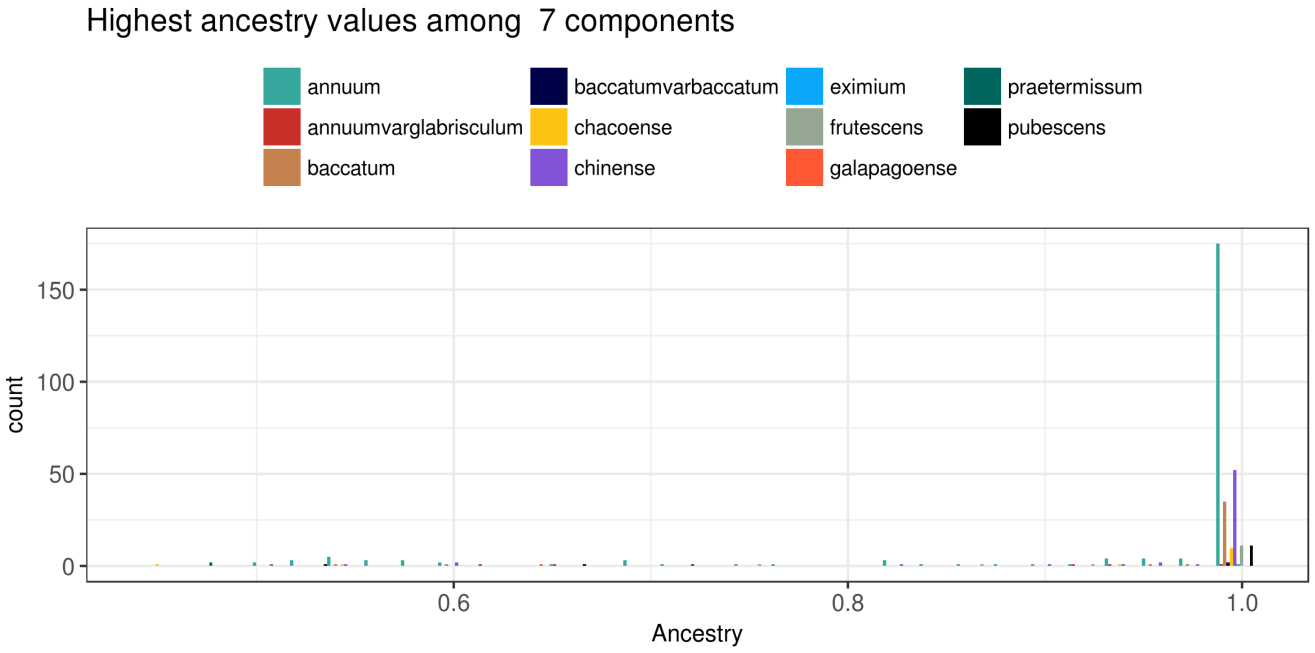

Figure S5. **Admixture results in the hypothesis of 7 clusters.** Accession have been grouped according to their geographical origin.

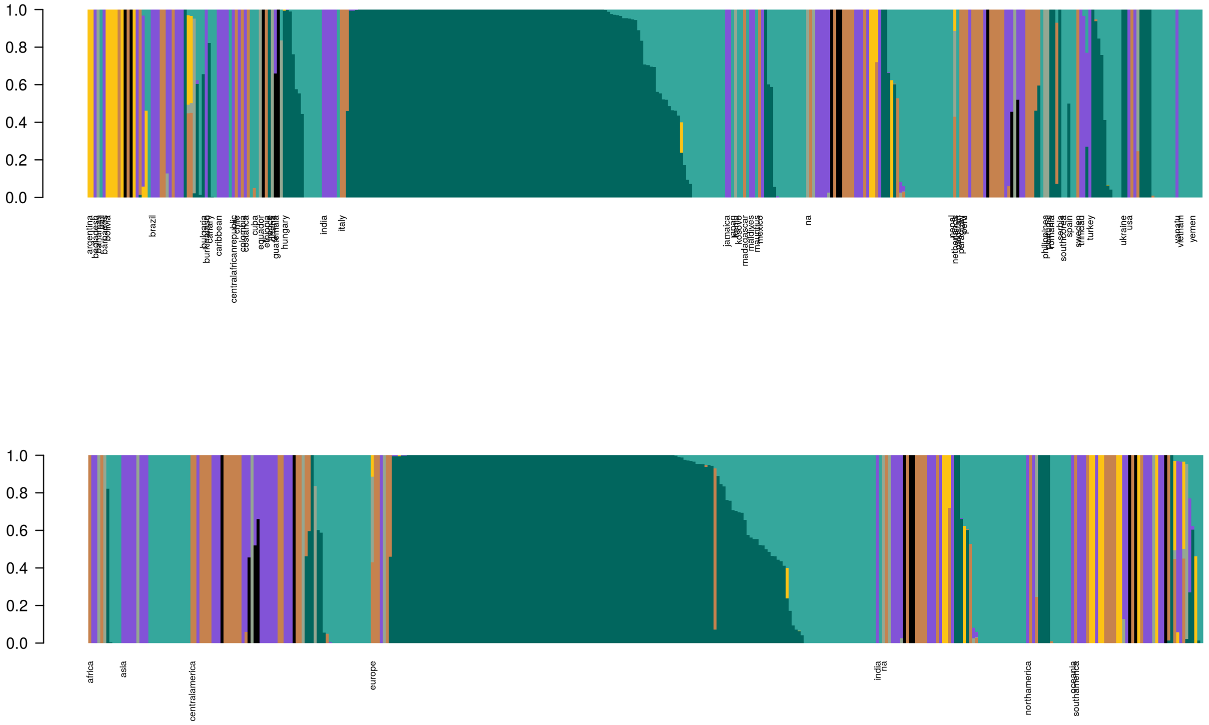

Figure S6. **Manhattan plots and QQplots for the phenotypes presenting significant associations.**

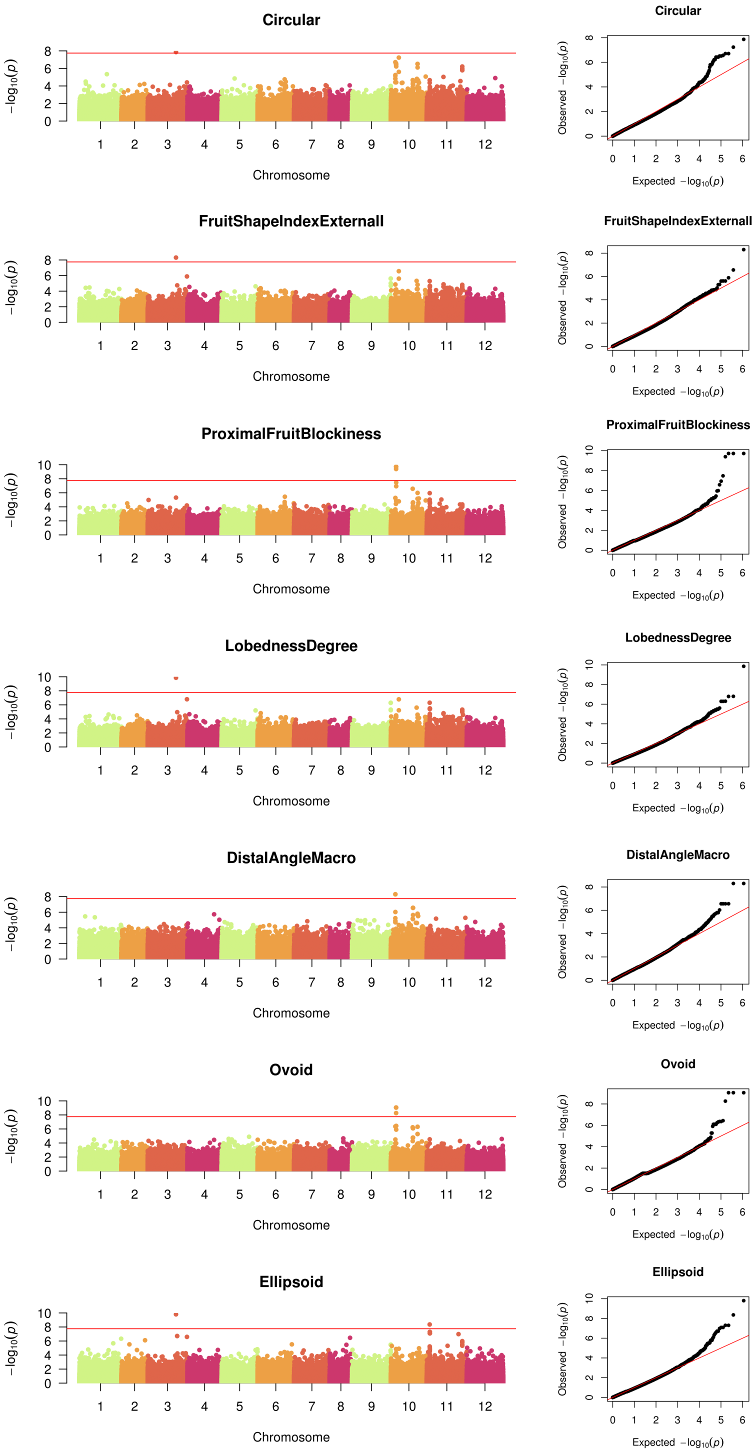

Figure S7. **PCA plots of 212 unadmixed Annuum samples.** PC1 visualizes the genetic variation between European and Non-european samples, PC2 visualizes the genetic variation in the European samples

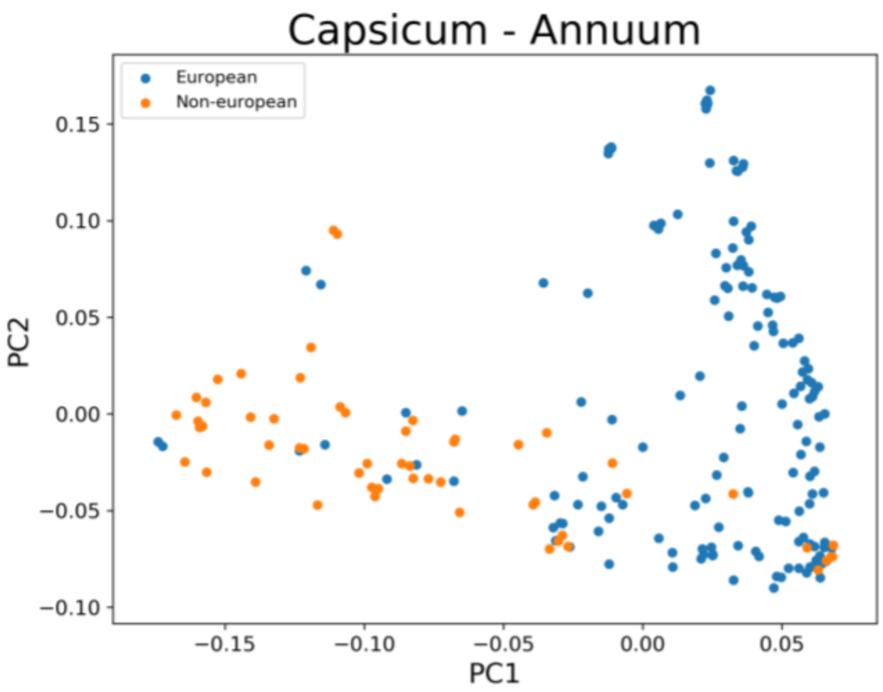

Figure S8. **PC2 selection scan.**

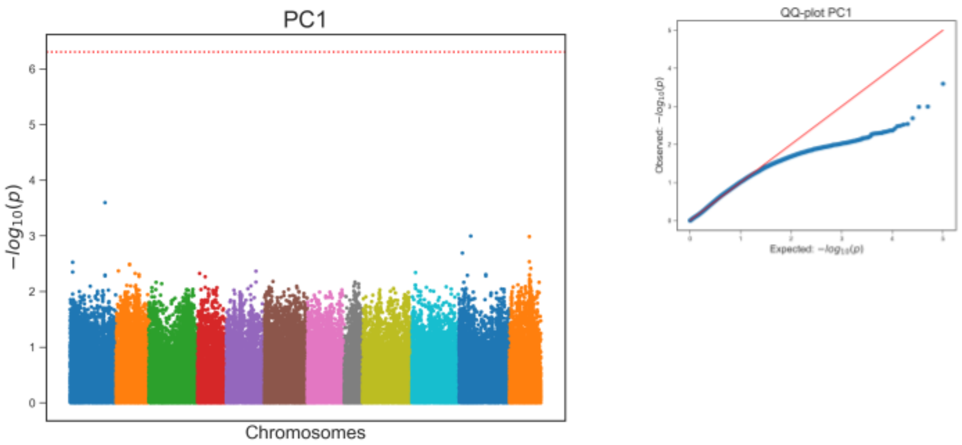

Figure S9. Contrasting phenotypes of fruits with genotypes CC and TT at the locus 3:183386147. The C allele is only found in the domesticated species. Fruits with the CC genotype tend to be elongated compared with fruits with the TT genotype irrespective of the overall fruit size. 1=Cornetto Giallo mirto crosia; 2=Ttaengcho; 3=Rogianiello; 4=Padron;5=Cayenna; 6=PI264281; 7=Tsilandilmila; 8=Uc Burun; 9=Vietnamese Red; 10=Corno di Toro Rosso; 11=Chiokiera; 12=Cornetto Piccante; 13=Quadrato d'Asti Giallo; 14=Marconi Giallo; 15=Mauritius; 16=Feherozon; 17=Soverato; 8=Sakaibe; 19=Boni; 20=Hungarian Orange.

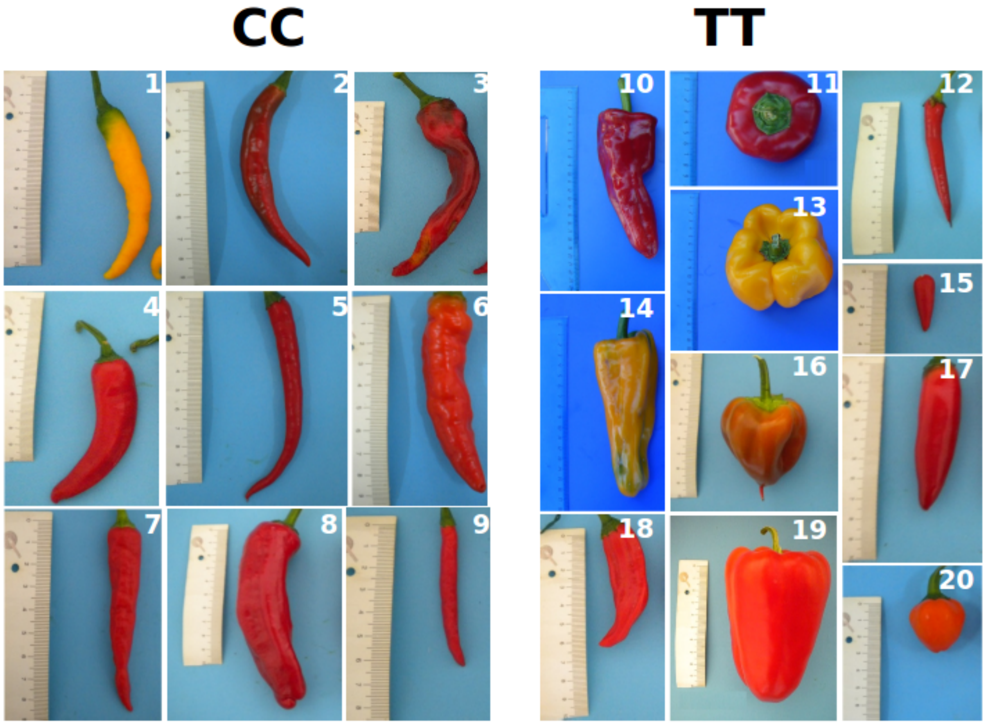

Figure S10. **Setup of the quantitative PCR.** (**a**)Standard curves for the markers LG1, LG2 and ACT2 using four dilution series and three replicates. (**b**) Reproducibility of results obtained with LG1 and LG2. I = Immature fruits, M = Mature fruits

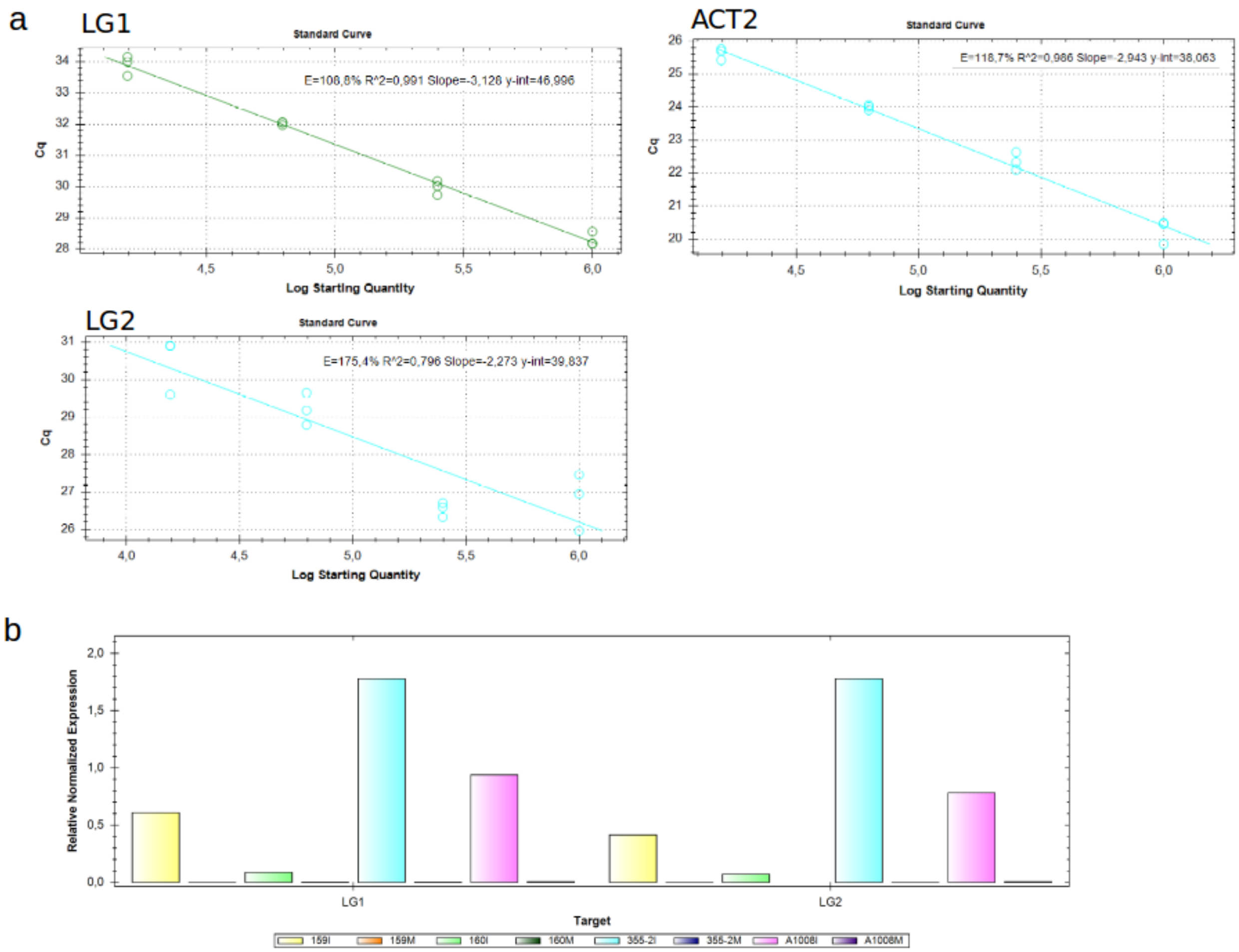

Additional file 2 — Supplementary Tables
